# Dynamical Analysis of Standing Balance Control on Sloped Surfaces in Individuals with Lumbar Disc Herniation

**DOI:** 10.1038/s41598-020-58455-z

**Published:** 2020-02-03

**Authors:** Jinping Li, Yang Zhang, Shasha Song, Ying Hou, Yigen Hong, Shouwei Yue, Ke Li

**Affiliations:** 1 Laboratory of Motor Control and Rehabilitation, Institute of Biomedical Engineering, School of Control Science and Engineering, Shandong University, Jinan, 250061 China; 2 Department of Physical Medicine and Rehabilitation, Qilu Hospital, Shandong University, Jinan, 250012 China; 3 Department of Rehabilitation Medicine, The Affiliated Suzhou Hospital of Nanjing Medical University, Suzhou, 215002 China

**Keywords:** Physical examination, Biomedical engineering

## Abstract

The changes of balance control mechanism caused by lumbar disc herniation (LDH) has not been well understood. This study aimed to investigate the effects of LDH on the balance control during standing on sloped surfaces. Ten patients with LDH and 10 gender- and age-matched healthy subjects were instructed to stand quietly on a sloped surface at −5°, 0° or +5°, respectively. The trajectories of the center of pressure (COP) of each individual limb and the full-body were recorded. Cross recurrence quantification analysis (CRQA) was applied to assess the coordination of COP components at the anterior-posterior and medial-lateral directions. The patients with LDH presented magnified inter-limb load asymmetry and had more deterministic components in the COP coordination of the less-affected limb and the full-body than the healthy subjects. The LDH led to decreased dynamical degree of freedom and less flexibility in bidirectional controlling the center of mass simultaneously. The effects of sensorimotor deficits due to LDH could be more obviously exhibited as standing on a declined rather than an inclined surface. This study shed light on the effects of LDH on standing balance control and may facilitate to develop novel strategies for evaluation of LDH.

## Introduction

Lumbar disc herniation (LDH) is a localized displacement of intervertebral disc tissue beyond the physiological margins of the intervertebral disc space. The prevalence of LDH is about 1–3%, mostly among the people aged between 30 and 50 years^1^. The symptoms of LDH include but are not limited to low back pain, leg pain, sciatica, muscle spasm or cramping, leg weakness, loss of leg function and abnormal gait^2,3^. Posture control during quiet standing could be vulnerable to the LDH. However, despite the accumulating evidence from the non-specific low back pain without structural change, inflammation or specific disease^4^, the potential effects of LDH on postural control for quiet standing are less studied.

To regulate balance is a preliminary request for the posture control during standing. When maintaining the body in balance, the sensory and motor systems need to be seamlessly integrated. The sensory information from visual, somatosensory and vestibular systems can facilitate the central nervous system to determine the orientation and state of body in environment^5^, make correct motion planning and issue suitable commands to the motor system^6^. The motor system, involving muscles, bones and joints, can generate a corrective, stabilizing torque to maintain the postural stability and orientation within the base of support^7^. This sensorimotor integration mechanism guarantees the fundamental requirement of balance control, and enables the body to respond to external disturbances in a timely and effective manner^8^. In LDH, deficits in the sensory system, such as reduced somatosensory sensitivity of foot sole^9^, lessened sensory nerve action potential^10^, abnormal H-reflex complex^11^ and in the motor system, such as decreased muscle strength of the trunk, knees, and ankles^12,13^, may potentially increase the sway of body and thus challenge the standing balance. More studies are deserved to specify the effects of LDH on the balance control during standing.

In contrast to the stable, horizontal surface, standing on a sloped surface further raises the difficulty of balance control, requiring promoted sensorimotor integration. The sensory system needs to provide more real-time sensory information about the altered perpendicular relationship between the support surface and gravitational vertical due to the slope; and the motor system should generate well-organized muscle activations and appropriate forces against the effects of slope. Examination of the balance control when standing on a sloped support surface may facilitate to clarify the relative contributions of the support surface and the gravity as reference frames for postural orientation^14–17^ and to detect the altered lower-limb muscle contraction and synergy for balance control^18,19^.

The balance control during standing can be approximated to a single inverted pendulum rotating around feet. The trajectory of center of pressure (COP) — the mean position of the forces acting under the feet at any instant in time, is strongly related to the movements of the body’s center of mass and can serve as an effective indicator for balance control. The COP trajectories are characterized by non-stationary, time- variation and complex temporal-spatial structures. Quantifying such temporal-spatial structures entails suitable analytical tools. Traditional time- or frequency domain approaches usually show limitations in analyzing the highly complex, nonlinear and nonstationary COP signals. Nonlinear dynamical analyses, such as the entropy, Lyapunov exponent or detrended fluctuation analysis, have been applied in assessment of the COP signals and helped to find out the dynamical patterns underlying the balance control in either the anterior-posterior (AP) and medial-lateral (ML) directions. Considering the posture should be controlled in both the AP and ML directions simultaneously during standing, it would be beneficial to quantify the dynamical coordination of the COPs in AP and ML directions. Recently, the cross recurrence quantification analysis (CRQA) has been developed as an advanced tool in assessment of dynamical coordination of nonlinear, nonstationary neurophysiological signals, and has been used in examination of the COP coordination between the AP and ML directions^20–22^. The measures derived from CRQA provide insights into the deterministic or stochastic components, structural complexity, periodic patterns, or synchronization underlying the dynamical COP coordination across the AP and ML directions. An intriguing issue is whether the CRQA parameters could reflect the potential changes in the bidirectional COP coordination as standing on a sloped surface, and disclose the LDH induced alterations in the highly fuzzy, complex, and dynamic balance control.

This study aimed to investigate the effects of LDH on the balance control during standing on a slope. The dynamical coordination of COP in the AP and ML directions were quantified using the CRQA. It is hypothesized that the patients with LDH would show more severe asymmetry of the load distribution between legs, and had more deterministic components in the COP coordination between the AP and ML directions than the healthy individuals.

## Methods

### Subjects

Ten patients with LDH and 10 gender- and age-matched healthy control (HC) subjects participated in the experiment. All the patients had been clinically diagnosed with LDH from the Department of Orthopedic, Qilu Hospital, Shandong University. The individuals who had history of cardiovascular, cerebrovascular or vestibular diseases, or musculoskeletal injuries on their lower-extremity were excluded. The patients received physical assessment of the symptoms and severity of LDH including the pressure and radiating pain, spasm, superficial sensation and muscle strength, and dysfunction assessment through three scales – Japanese Orthopaedic Association Scores (JOA), Oswestry Disability Index (ODI) and Roland Morris Disability Questionnaire (RMDQ) from the Department of Physical Medical and Rehabilitation, Qilu Hospital, Shandong University. The more- and less-affected limb of LDH patients were defined by above-mentioned physical assessment. If one leg had more serious symptoms compared with the other leg, it was marked as the more-affected limb. Meanwhile, the other was the less-affected limb. The characteristics of subjects are shown in Table 1. The study was carried out in accordance with the 1964 Helsinki declaration and its later amendments or comparable ethical standards. All participants provided their written informed consent following the protocols approved by the Institutional Review Board of Shandong University.

**Table 1:** Characteristics of subjects.

	LDH							HC
Number	Sex	Age (y)	Level of herniation^a^	JOA^b^	ODI^c^	RMDQ^d^	More-affected limb	Age (y)
**1**	Female	56	L4-L5, L5-S1	18	16	12	Left	55
**2**	Male	50	L4-L5	10	32	22	Left	49
**3**	Female	35	L5-S1	15	23	17	Left	32
**4**	Female	69	L5-S1	15	24	17	Right	68
**5**	Male	43	L4-L5, L5-S1	14	19	9	Left	44
**6**	Female	61	L3-L4, L4-L5	10	23	15	Left	62
**7**	Male	45	L5-S1	16	26	13	Right	45
**8**	Male	65	L4-L5	13	28	18	Right	64
**9**	Female	39	L4-L5	16	10	6	Right	42
**10**	Female	45	L5-S1	15	16	11	Left	44
**Mean**		50.80		14.20	21.70	14.00		50.50
**SD**		11.46		2.57	6.52	4.74		11.41

^a^L: Lumbar spine; S: Sacrum; ^b^JOA: Japanese Orthopaedic Association Scores; ^c^ODI: Oswestry Disability Index; ^d^RMDQ: Roland Morris Disability Questionnaire.

### Experimental set-up

A force platform (FDM-S, Zebris Medical GmbH, Isny, Germany) covered with 64*40 pressure sensors was used to measure the foot plantar pressure distributions at a sampling frequency of 60 Hz. The COP time series were processed in the MATLAB R2017b (The Mathworks, Natick, MA, USA). Parameters of CRQA were implemented with the cross recurrence plot toolbox 5.16^23^.

### Test procedures

**Figure 1:**
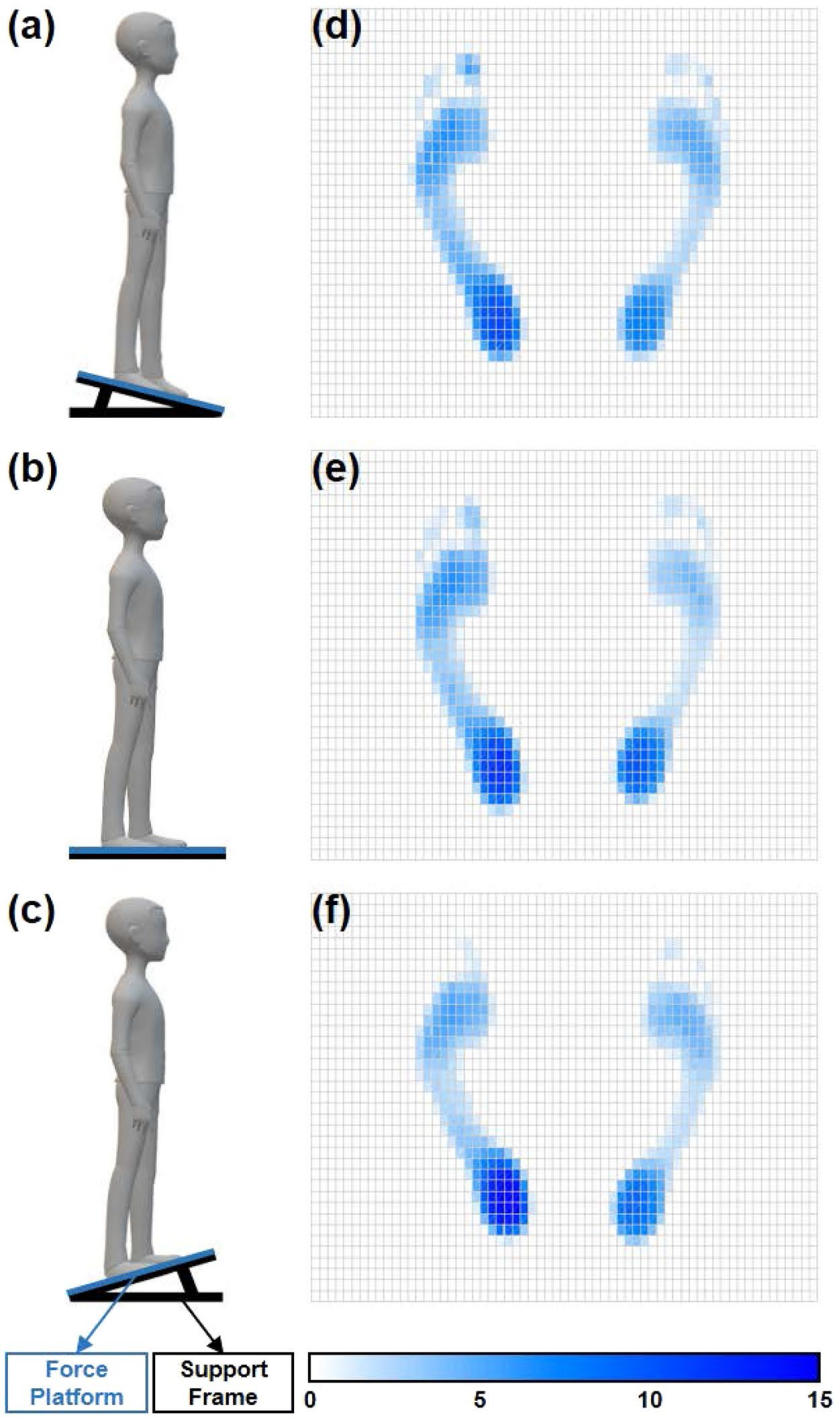
The schematic diagram of the experiment set-up and foot plantar pressure distributions from a representative patient with LDH. (**a**) Declined surface at −5°; (**b**) horizontal surface at 0°; (**c**) inclined surface at +5°; (**d**), (**e**) and (**f**) are foot plantar pressure distributions corresponding to (**a**), (**b**), (**c**), respectively.

The schematic diagram of the experiment set-up is depicted in Fig. 1. Subjects were required to stand barefoot quietly on the center of the force platform for 30 s, with their feet side by side, their hands naturally on both sides of body and their eyes gaged forward. Three angles of the force platform surface, including −5°, 0° and +5° with respect to the horizontal plane, were tested with a random sequence. For each surface angle, only one trial was performed by each subject. A 5-min rest was given between trials. In order to avoid the influence of free body swing, the data for the first and last 5 s was removed, leaving the middle 20 s for the further analysis.

### Data analysis

In order to explore whether patients with LDH shifted the load from the more-affected limb to the less-affected limb, we calculated the limb load asymmetry (LLA), which was defined as the ratio of the loads on the less-affected to on the more-affected limb^16,22,24,25^:

1 $$LLA=\frac{Load\,on\,{Less}-{affected}\,Limb}{Load\,on\,More-affected\,Limb}$$

For the healthy individuals, the more- or less-affected limbs were determined in accordance with their counterpart patients with LDH^26^.

The COP was calculated from the signals recorded by each pressure sensor as follows:

2 $${{\rm A}}{{\rm P}}=\frac{\sum {F}_{i}\ast {Y}_{i}}{\sum {F}_{i}}$$

3 $$ML=\frac{\sum {F}_{i}\ast {X}_{i}}{\sum {F}_{i}}$$

where the *AP* and *ML* represent the COP coordinates at one moment in the AP and ML directions, respectively; *i* is the number of pressure sensors, *F*_*i*_ is the force on the *i*^*th*^ unit area, and (*X*_*i*_, *Y*_*i*_) is the coordinate of the *i*^*th*^ sensor. The COP of the more-affected limb (COP_M_), less-affected limb (COP_L_) and the full-body (COP_net_) were computed according to the formula (2) and (3). Figure 1 shows the distributions of foot plantar pressure at a moment from a representative patient with LDH as standing on the force platform at different angles.

In order to quantify the characteristics of dynamical coordination between AP and ML directions, the COP time series in the two directions need to be reconstructed and projected into a phase space with appropriate parameters. The time delay and embedding dimension, two parameters essential for phase-space reconstruction, were determined by mutual information and false nearest neighbors in this study. Eventually, the time delay at 6 samples and the embedding at 4 were determined and used in the following CRQA analysis. A window with 600 sample points and an overlap of 300 sample points were applied on the signal series to quantify the characteristics of the dynamical coordination for COP trajectories. The mean values of all the windows were calculated for each subject.

The dynamical coordination of COP in AP and ML directions were further quantified by the CRQA, which provides a quantification of a cross recurrence matrix (CR):

4 $$C{R}_{i,j}^{\tau ,m}=\varTheta (\varepsilon -\Vert u(i)-v(j)\Vert )$$

where the *u*(*i*) and *v*(*j*) are the phase-space vectors corresponding to COP series in AP and ML directions; *i*, *j* = 1, …, *N*, *N* is the length of COP series; *τ* and *m* are the predefined time delay and embedding dimension, respectively; *ε* is the threshold and was set to 30% of the maxim phase space radius. The *Θ* is the Heaviside function and || || is the Euclidean norm. The CR contains all the recurrence states between two trajectories of time series reconstructed in the phase space. A visualization of CR is cross recurrence plot (CRP). We used the cross recurrence rate (X-RR), cross determinism (X-DET) and cross entropy (X-ENT) to quantify the structure of the CRP. The X-RR is defined as:

5 $${X} \mbox{-} {RR}=\frac{1}{{N}^{2}}\mathop{\sum }\limits_{i,j=1}^{N}C{R}_{i,j}$$

where the X-RR indicates the regularity by measuring the density of recurrence points in the CRP^23^. The greater X-RR indicates greater correlation between the COP series in the AP and ML directions. The X-DET is defined as:

6 $${X} \mbox{-} {DET}=\frac{{\sum }_{l={l}_{{\min }}}^{N}l\,\ast \,P(l)}{{\sum }_{l=1}^{N}l\ast P(l)}$$

where *l* is the diagonal sequence of recurrence points, *l*_*min*_ is the least length, equaling to 2; *P*(*l*) is the frequency distribution of the lengths of the diagonal lines. The X-DET is the ratio of recurrence points that form diagonal structures to all recurrence points, relating to deterministic of dynamical system^23^. The X-ENT is defined as:

7 $$X \mbox{-} ENT=-\mathop{\sum }\limits_{l={l}_{{\min }}}^{N}p({l})\ast \mathrm{ln}(p({l}))$$

where *p*(*l*) is the probability of a diagonal line, estimated from *P*(*l*)^23^. The X-ENT reflects the complexity of the deterministic structures in the dynamical coupling of COP^23^.

All statistical analyses were performed using SPSS 23.0 (SPSS Inc., Chicago, IL). Kolmogorov-Smirnov test was used to examine the data distribution. A two-way repeated measures ANOVA was applied to examine the differences of the CRQA parameters for the COP_L_, COP_M_ and COP_net_, with the groups (LDH vs. HC) as between-subject factor and angles (−5°, 0°, +5°) as within-subject factor. The Huynh-Feldt correction was used when the assumption of sphericity was violated. Post-hoc multiple pairwise comparisons were corrected by Bonferroni correction. An independent *t*-test was applied to examine the differences between the LDH and HC groups. A *p*-value less than 0.05 was considered as statistical significant.

## Results

**Figure 2:**
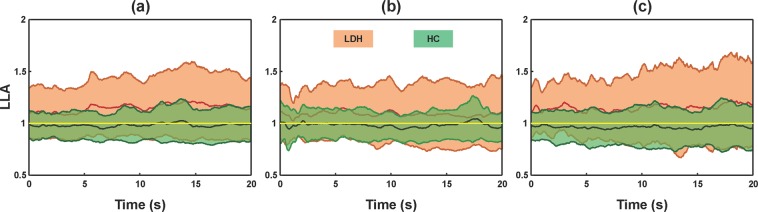
The mean and standard deviations of the time series of the limb load asymmetry (LLA) for all the patients with LDH and the healthy subjects (HC). (**a**) Declined surface at −5°; (**b**) horizontal surface at 0°; (**c**) inclined surface at +5°.

The time series of LLA are depicted in Fig. 2. Different from the healthy subjects whose LLAs were close to 1 (−5°: 0.982 ± 0.147; 0°: 0.982 ± 0.137; +5°: 0.962 ± 0.178), the LDH patients showed LLAs much higher than 1 with greater standard deviations (−5°: 1.152 ± 0.284; 0°: 1.086 ± 0.282; +5°: 1.151 ± 0.332). In addition, the LDH group had higher LLA values at sloped surfaces compared with that at 0°.

**Figure 3:**
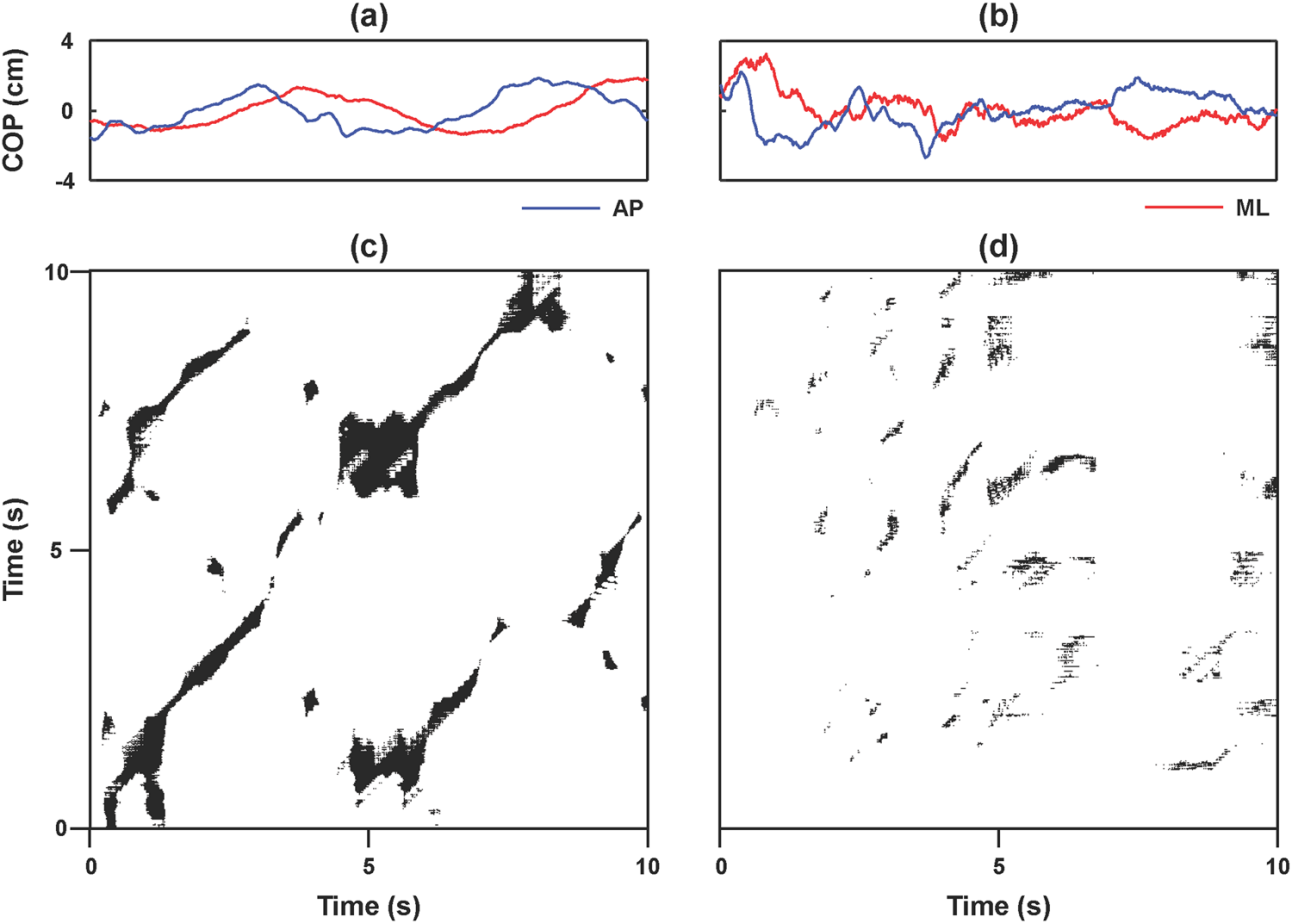
The full-body COP trajectories and the cross recurrence plots (CRP) during standing on a horizontal surface. (**a**) The COP components of the anterior-posterior (AP) direction and the medial-lateral (ML) directions from a representative patient with LDH; (**b**) The COP components of the AP and the ML directions from a healthy subject; (**c**) The CRP of (**a**); (**d**) The CRP of (**b**).

Figure 3 exhibits the representative COP_net_ trajectories in the AP and ML directions and the CRPs from a patient with LDH and a healthy subject when standing on the force platform. Compared with the healthy subject, the LDH patient had more recurrence points and linear structures along the diagonal in the CRP, reflecting more regular and deterministic structures in coordinating the COP components between the AP and ML directions when maintaining the standing balance.

**Figure 4:**
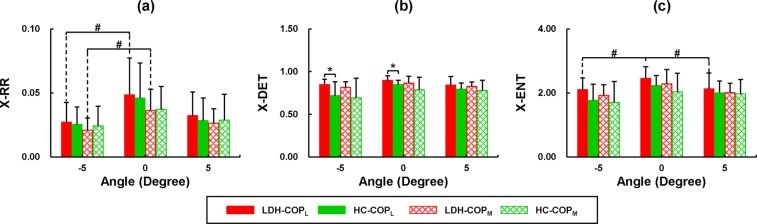
Statistical results of the CRQA analysis for the less- and more-affected limbs. ^*^Significant difference between the LDH and HC groups (*p* < 0.05); ^#^significant difference across the slope angles (*p* < 0.05).

Results of the CRQA for COP of each limb are shown in Fig. 4. The X-DET of COP_L_ showed significant differences between groups (*F*_*1,18*_ = 5.722, *p* = 0.028): the patients with LDH showed significantly higher X-DET of COP_L_ than the healthy individuals at −5° (*t* = 2.368, *p* = 0.029) and 0° (*t* = 2.105, *p* = 0.050, Fig. 4b). Effects of slope angles were only observed in the CRQA parameters of the LDH group, rather than the healthy subjects. Specifically, the X-RRs of the COP_L_ and COP_M_ of the LDH patients at 0° were significantly higher than those at −5° (COP_L_: *p* = 0.027, COP_M_: *p* = 0.023, Fig. 4a). No significant difference of the X-RRs was observed between the 0° and +5°. Neither, no significant difference was found between angles for the X-DET. With respect to the horizontal plane, significantly lower X-ENT of COP_L_ was found on −5° (*p* = 0.012) and +5° (*p* = 0.005, Fig. 4c) slopes. By contrast, no significant difference was found between the angles for the X-ENT of COP_M_.

**Figure 5:**
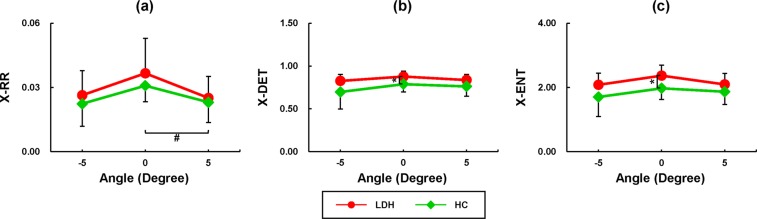
Statistical results of CRQA analysis of the full-body COP. ^*^Significant difference between the LDH and HC groups (*p* < 0.05); ^#^significant difference across the slope angles (*p* < 0.05).

Figure 5 shows the results of CRQA for the COP_net_. Significant differences between the LDH and HC groups were observed in the X-DET (*F*_*1,18*_ = 4.964, *p* = 0.039) and X-ENT (*F*_*1,18*_ = 4.451, *p* = 0.049). The patients with LDH exhibited significantly higher X-DET (*t* = 2.532, *p* = 0.021, Fig. 5b) and higher X-ENT (*t* = 2.623, *p* = 0.017, Fig. 5c) than the healthy subjects at 0°. No significant difference was observed between the two groups in the X-RR. Effects of slopes were only found in the X-RR of HC group (*F*_*2,36*_ = 8.244, *p* = 0.001), showing significantly lower X-RR at +5° than 0° (*p* = 0.017, Fig. 5a).

## Discussion

This study investigated the effects of LDH on the balance control during standing on sloped surfaces. The load asymmetry was quantified using LLA; and the dynamical coordination of COP components between the AP and ML directions was analyzed for each limb and the full-body using the X-RR, X-DET and X-ENT of CRQA.

The results of LLA indicated augmented asymmetry in the load distribution between legs in LDH (Fig. 3). Compared with the healthy subjects, the patients with LDH showed much higher LLA, implying that their less-affected limb bore more weight than the more-affected limb. These results corroborate with the previous findings that the degree of lower back pain correlated with the weight-bearing asymmetry between legs^27^. The results of the current study suggest that the patients with LDH prefer to transfer the load from the more-affected limb to the less-affected limb, thereby magnifying their inter-limb asymmetry during standing. In addition, the LLA of the patients showed much higher standard deviations than that of the healthy subjects, suggesting a greater variations in the load distributions between the two legs due to LDH. Clinical observations suggest that people with lower back pain performed more body weight shifts to reduce the discomfort^28,29^. It is noteworthy that when standing on a sloped surface, the patients with LDH, rather than the healthy subjects, showed higher but more variant LLA. These results suggest that standing on a sloped surface could facilitate to present the effects of LDH, specifically, the augmented load-distribution asymmetry and load-sharing variation between legs.

The effects of LDH on the COP coordination between the AP and ML directions were observable from the X-DET of the less-affected limb (Fig. 4b). On the 0° and −5° surfaces, the X-DET of COP_L_ in LDH group was higher than that of HC group for the less-affected leg. The increased X-DET in LDH indicates more regular and deterministic temporal structures in coordinating the COP between the AP and ML directions than the healthy subjects, revealing decreased dynamical degree of freedom, limited adaptability to potential external perturbations, and increased restriction on balance control due to the LDH. The LDH-induced X-DET increase was only observed from the COP_L_, suggesting a decreased flexibility in the dynamical COP coordination between the AP and ML directions on the less-affected leg, which is consistent with the previous findings that the extremity bearing heavier load presented strengthened COP coupling^30^. In addition, previous studies suggested that the less-loaded foot could compensate for the loss of flexibility of the more-loaded foot during standing^31^. But in the current study, although the more-affected leg were less loaded than the less-affected leg in LDH, no significant difference of COP_M_ was observed in the X-DET between LDH and HC groups, revealing a limited shift of the flexibility in the COP coordination between two legs due to LDH.

Patients with LDH also showed increased X-DET and X-ENT in the COP_net_ than the healthy subjects (Fig. 5). The higher X-ENT indicated increased complexity of the deterministic structures in the dynamical coupling of COP^23^. This result suggests that the difference in flexibility of the single-leg COP coordination between the LDH and HC groups may also influence their full-body COP coordination. The increased X-DET and X-ENT of the full-body COP coordination revealed a decreased dynamical degree of freedom, limited adaptability to the potential external perturbations, and less flexibility in controlling the center of mass in the AP and ML directions simultaneously. These changes would be highly related to the sensorimotor impairments induced by the LDH. As a disc herniates and spills into the spinal canal, the compression of the spinal cord or the nearby nerve roots could debilitate the sensorimotor function of the lower extremity^9,11,32^. Standing is charged by a complex closed-loop feedback control mechanism, which integrates the sensory information from visual, vestibular and somatosensory systems and generates appropriate muscle forces and body orientations to maintain balance. The sensorimotor deficits due to LDH could interfere with this control mechanism by disturbing the detection and processing of the real-time sensory information, and weakening the muscle force production and coordination that are necessary for prompt and effective balance control. To counter the effects of sensorimotor deficits, the central nervous system would rely more on a feedforward mechanism based on default modes, sensorimotor memories or experiences, rather than the close-loop feedback control strategy^33,34^. The reinforced feedforward mechanism can compensate for the LDH-induced sensorimotor deficits, rendering stronger coupling and more deterministic structures of the full-body COP coordination. This would be in line with the previous findings that sensorimotor deficits due to aging or low back pain could result to decreased complexity of postural sway^33^ and reduced flexibility in postural control^35^.

Results further showed that the X-RR and X-ENT of the COP coordination on each leg (COP_L_ and COP_M_ in Fig. 4), and the X-RR of the full-body COP (COP_net_ in Fig. 5a) were significantly decreased when standing on sloped surfaces than on the horizontal surface, especially in the patients with LDH. The lower X-RR was related to decreased correlation between the COP components at the AP and ML directions; and the lower X-ENT indicated decreased complexity of the deterministic structures in the dynamical coupling of the AP- and ML-COP components^23^. In a recent study, Dutt-Mazumder *et al*.^36^ examined the COP of healthy subjects as standing on surfaces at different angles including +20°, +10°, 0°, −10°, −20°, −30°, −35°, and found that the COP dynamics on a flat surface were recurrent with an augmented deterministic process and higher Shannon entropy compared to elevated slope angles in dorsiflexion and plantarflexion. Consistent with this finding, the results of the current study further pointed out that standing on a sloped surface could result in increased randomness but decreased complexity in the directional coupling of COP compared with the horizontal surface. The changes in bidirectional coupling of COP could be associated with increased postural instability^37,38^ and direction-specific sway^38^, and would be resultant from the alteration of reference frame regarding gravity, support surface and posture orientation^16,17^, or the different muscle activations and synergies contributing to ankle stiffness^18,39^.

It would be noteworthy that the differences between the LDH and HC groups were observed in X-DET at −5° and 0°. In addition, the patients with LDH showed lower X-RR and X-ENT values at −5° with respect to 0°. These results partially confirmed our hypothesis that standing on a slope could facilitate to present the effects of LDH on the balance control by decreasing the dynamical degree of freedom, confining the adaptability and reducing the flexibility in the AP and ML directions simultaneously. Standing on a sloped surface would cause greater challenges in controlling the center of mass, particularly with the deteriorated sensorimotor function in LDH. In addition, previous studies found that the LDH can result in lower ankle dorsiflexor strength and make patients show a foot-drop gait^13^. The declined surface at −5° could thus intensify the deficiency in ankle dorsiflexion, whereas the inclined surface at +5° may effectively counteract this effect. This would help to interpret the difference in X-DET observed in the declined rather than the inclined surface.

This study may have some limitations. The magnitudes of pain were not precisely evaluated in the experiment. Therefore, we could not achieve quantitative assessment of the effects of pain/discomfort due to LDH on the postural stability. Moreover, the limited number of subjects did not allow us to explore the relationship between the magnitude of pain and postural stability. More subjects with accurately evaluated pain should be examined in order to clarify the effects of pain or discomfort arising from the LDH on the postural stability in future.

## Conclusions

This study investigated the effects of LDH on the balance control during standing on sloped surfaces. Results showed that the patients with LDH preferred to transfer the load from the more-affected limb to the less-affected limb, and thus magnified their inter-limb asymmetry during standing. The LDH led to decreased dynamical degree of freedom, limited adaptability to potential external perturbations, and less flexibility in controlling the center of mass in the AP and ML directions simultaneously. Compared with the horizontal surface, standing on a sloped surface could result in increased randomness but decreased flexibility in the bidirectional coupling of COP, suggesting an weakened feedback mechanism but a reinforced feedforward mechanism underlying the balance control. This study shed light on the effects of LDH on standing balance control and may facilitate to develop novel strategies for evaluation of LDH.

## Data Availability

The datasets are available from the corresponding author on reasonable request.
